# Association between social capital and health-related quality of life among left behind and not left behind older people in rural China

**DOI:** 10.1186/s12877-017-0679-x

**Published:** 2017-12-16

**Authors:** Yaqin Zhong, Pär Schön, Bo Burström, Kristina Burström

**Affiliations:** 1 School of Public Health, Nantong University, 9 Seyuan Road, Nantong, Jiangsu 226019 China; 2 Health Outcomes and Economic Evaluation Research Group, Stockholm Centre for Healthcare Ethics, Department of Learning, Informatics, Management and Ethics, Karolinska Institutet, Stockholm, Sweden; 3 Aging Research Center, Karolinska Institutet and Stockholm University, Stockholm, Sweden; 4 Equity and Health Policy Research Group, Department of Public Health Sciences, Karolinska Institutet, Stockholm, Sweden

**Keywords:** Social capital, Health-related quality of life, Left behind, Older people, Rural China

## Abstract

**Background:**

The association between social capital and health-related quality of life (HRQoL) has not been thoroughly studied among older persons in rural China, especially among those who were left behind or not. This study investigates the association between social capital and HRQoL and examines possible differences of this association between being left behind or not in rural China.

**Methods:**

A cross-sectional survey of 825 people aged 60 years and older, residing in three rural counties in Jiangsu Province in China, was conducted in 2013. Factor analysis was performed to measure social capital. EQ-5D was used to measure HRQoL. Tobit regression analysis with upper censoring was conducted to explore the association between social capital and EQ-5D index.

**Results:**

After controlling for individual characteristics, low social capital and being left behind were significantly associated with low HRQoL. Old people with low social capital had 0.055 lower EQ-5D index compared to those with high social capital. Old people being left behind had 0.040 lower EQ-5D index compared to those who were not left behind. For different dimensions of social capital, the main effects came from the domain of *trust and reciprocity*. There was a significant interaction between low social capital and being left behind on HRQoL, suggesting that low social capital was associated with low HRQoL among persons left behind.

**Conclusions:**

Our findings indicate that the left behind old people with low social capital were a potentially vulnerable group in rural China. Formulating and implementing initiatives and strategies which increase social capital may foster better HRQoL, especially for old people who were left behind.

**Electronic supplementary material:**

The online version of this article (doi: 10.1186/s12877-017-0679-x) contains supplementary material, which is available to authorized users.

## Background

### Health-related quality of life among older adults

Population aging is a global phenomenon and has become a huge challenge for most societies across the world. Compared to developed countries, the pace of aging is much faster in developing countries [1]. Over the past two decades, China’s population has been aging rapidly. According to statistics, more than 202 million people were over 60 years old in 2013 and this number is set to reach about 288 million by 2025, and 370 million by 2050 [2]. In rural China, 117 million Chinese were older than 60 years and accounted for 17% of the total rural population in 2012 [3].

Health-related quality of life (HRQoL) is a commonly used measure of health outcome. It reflects several dimensions of health, including physical, psychological, social, cognitive function, as well as general well-being [4]. HRQoL is associated with a wide range of factors, including biological, psychological and social factors. Advanced age is associated with increasing health problems, which means a decline in HRQoL. Lower education, poor economic situation are also associated with low HRQoL [5]. Previous studies have shown that low HRQoL is common among those being left behind [6, 7]. In China, with the process of urbanization and industrialization, many young adults migrate to cities in search of work and their older parents are left behind.

Over the past two decades, there has been increasing attention paid to social capital as an important influencing factor of HRQoL [8–10]. Previous studies have shown that low social capital is associated with lower HRQoL [8–10]. Maintaining a high level of HRQoL into advanced age is an increasingly important public health issue, both from an individual and societal perspective.

### Theoretical frameworks of the social capital concept

Social capital has increasingly gained recognition as an important concept in public health. In regard to aging, it has been pointed out that high levels of social capital enables older adults to maintain independent, productive and satisfactory lives [8, 11]. Though many studies have focused on social capital, it is a challenge to define and investigate this multidimensional concept [5, 12–14]. According to Bourdieu, social capital is “the aggregate of the actual or potential resources which are linked to possession of a durable network” [5]. Coleman pointed out “not a single entity, but a variety of different entities having two characteristics in common: they all consist of some aspect of social structure, and they facilitate certain actions of individuals who are within the structure” [12]. While Putnam defined social capital as “features of social organization, such as trust, norms and network, that can improve the efficacy of society by facilitating coordinated actions” [13]. Bourdieu and Coleman’s views are typical for a “network view” while Putnam emphasizes “social cohesion”. In this study, we argue an operational definition of social capital, which can be defined as “the structured social networks, trusting relationships and cultural norms that act as resources for individuals and facilitate collective action” [15].

Since the publication of previous theories, most researchers have distinguished structural and cognitive dimensions of social capital [11, 16, 17]. The former subsumes attitudinal manifestations, such as values, norms, beliefs while the latter refer to the aspects of social organization such as social participation or social networks [16]. In this study, we also use this framework to choose the indicators of social capital.

Measurements of social capital vary among countries, regions, researchers and different populations [11, 18, 19]. There is no consensus about which methods are the most appropriate to measure this multidimensional concept [20]. The measurements should also consider cultural factors and the characteristics of the society. Sun performed factor analysis to extract social capital factors, which included social participation, reciprocity and social support, trust and safety, interpersonal relationship network and neighborhood cohesion [10]. Yip used the number of organizations to which the respondent belongs as the structural social capital and “trust” as the cognitive social capital [16]. Following literatures and similar studies in China [10, 16], some commonly used measures such as social participation, trust, personal networks are taken as proxy measures in our study [13].

### Social capital and health-related quality of life

It has been argued that social capital is a determinant for good health in older ages, contributing to better health outcomes including HRQoL [21]. Studies focused on the older people have found that high social capital is associated with good HRQoL, even after controlling for relevant confounders [9, 22]. The possible mechanisms include (1) influence on the individuals’ health-related behaviors, (2) influence on access to amenities and services and (3) influence on psychosocial processes [10].

Most studies on social capital and HRQoL have been conducted in high income countries, however, the association has not been thoroughly studied in China, especially for older people who are left behind. Several studies from China have explored the relationship between social capital and health outcomes among older adults. Sun et al. (2015) used SF-36 to measure HRQoL and revealed a positive association between individual social capital and HRQoL in rural China [10]. Yip and colleagues (2007) found that cognitive social capital was positively associated with health outcomes including self-rated health, psychological health and subjective well-being, while there was little association between structural social capital and these outcomes variables [16]. To the best of our knowledge, none of the above mentioned studies have studied the group of older people being left behind, even though being left behind may be an important determinant of HRQoL.

### Being left behind and health-related quality of life

According to statistics from the Ministry of Civil Affairs of China, around 50 million older people were left behind in rural China by 2015 [23]. Previous studies on how children’s migration affects the health of their left-behind parents are ambiguous. On the one hand, children’s migration can improve their parents’ financial situation and allow the left-behind parents to get better nutrition and health care; on the other hand, the migration also reduces the social support from children and increases risks of depression and other adverse health outcomes [7, 24]. Even though there are some studies concerning this issue, they have not made consistent conclusions. Given that social capital and being left behind may be important and growing determinants in health policy, health-related interventions should be targeted towards the groups who are in the greatest need.

The general aim of the present study was to investigate the association between HRQoL and social capital among older people in rural China. The specific research questions are: what is the association between social capital and HRQoL among older adults in rural China? What is the difference of HRQoL between those who are left behind or not? What is the difference of the association between social capital and HRQoL between those being left behind or not?

## Methods

### Sampling and data collection

The data used in this study came from a cross-sectional survey conducted in three rural counties in Jiangsu Province in China between July and August in 2013. These three rural counties are located in Suzhou, Nantong and Yancheng City, respectively. The areas were chosen to represent different geographic and socioeconomic distribution. In each county, two towns were randomly selected and in each town, three villages were also randomly selected. Within each village, about 50 households with a person aged 60 years and above were randomly selected. If the respondents could not read or write, the trained interviewers from Nantong University introduced the questions to them and then filled out the answers. A total of 825 old individuals completed the survey. The response rate was 95%.

The survey collected data on individual characteristics such as age, sex, marital status, educational level, a variety of social capital indicators and HRQoL measured by the EQ-5D instrument.

Before the survey, the interviewers introduced the survey to the respondents. They were informed about the aim of the survey, the selection criterion of the sample, and the assurance that the information was only used for research and got their informed consent.

### Dependent variable

The dependent variable in the present study is EQ-5D. As one of the most world-widely used instruments for measuring HRQoL, the EQ-5D-3 L defines HRQoL in five dimensions (mobility, self-care, usual activities, pain/discomfort, and anxiety/depression) with three levels of severity (no problems, moderate problems and severe problems). In total 243 health states or health profiles can be generated by this classification [25] and each of them can get an index value based on a value set where full health = 1 and dead = 0. The EQ-5D is simple and feasible for surveys aimed at older population with low education [26]. In the present study, the value set by Liu and Wu (2014) was used to obtain the EQ-5D-3 L index [27].

### Independent variables

The independent variable in the present study is social capital. Some previous studies only use secondary data and did not design their own measures of social capital [15, 28, 29]. In other studies, comprehensive measures have been designed but these measures were a small part of the household survey [29]. A questionnaire survey was designed to assess the respondents’ social capital in our study. Some researchers pointed out cultural factors are important to determine social capital [30, 31]. So we selected fourteen commonly used questions and adapted to the Chinese context (see Additional file 1). An exploratory factor analysis was conducted to investigate the factor patterns of different dimensions of social capital. For structural social capital, questions such as the quantity and frequency of organization participation, the number of relatives and friends and the occupations of them, relationships and mutual aid between neighborhoods were included. For cognitive social capital, the questions whether the respondents trust in villagers or leaders in the village were included to measure the dimension of “trust”. Subsequently, with the results from the factor analysis, we calculated the score of social capital and then explored the association between social capital and HRQoL.

Age was categorized into 60-69 years (reference group), 70-79 years and 80 years and above. Being left behind was defined as older individuals with adult children leaving for more than six months during the past year; not being left behind was reference group [32]. Marital status was divided into married (reference group) and others, which including single, widowed and divorced. Educational level was also controlled, which was divided into primary school and above (reference group) and illiterate.

### Statistical analyses

Descriptive statistics were expressed as mean ± standard deviation. Factor analysis was performed to extract social capital dimensions (using Eigenvalue > 1 as the selection criterion) [33]. Then the score of social capital was calculated and dichotomized into low (lowest 25% of the sample) or high (> 25%) social capital.

EQ-5D index is a censored variable, for example, a great number of respondents had the index of 1 in our study. The Tobit regression model was considered suitable for bounded or censored data [34].

In the present study, three regression models were run. In Model 1, all controls were included, including sex, age, marital status, educational level, social capital and being left behind or not. Model 2 included individual characteristics and all the distinct social capital dimensions. Model 3 controlled for individual characteristics and interactions pertaining to their left-behind status. Coefficients and standard errors were used to compare the effect on the EQ-5D index.

To consider the possible differences between those who were left behind or not, a stratified analysis was also carried out among those left behind and those not left behind. All analyses were conducted using Stata 14.0; a 5% significance level was used.

## Results

### Individual characteristics of the sample

The distribution of the individual characteristics is presented in Table 1, stratified by being left behind and not being left behind. More than half (54%) of the respondents were female. About half (51%) was aged between 60 to 69 years old. The majority were married (73%) at the time being interviewed. Nearly half of the respondents (46%) were illiterate. The proportion being married was higher among those left behind compared to those not left behind. As to sex, age and marital status, there were no differences between those left behind and those not left behind.

**Table 1:** Characteristics of the respondents and problems in EQ-5D dimensions by being left behind and not being left behind (*n* = 825)

Variables	Total *n* (%)	Left behind *n* (%)	Not left behind *n* (%)	*P* value
Sex				
Male	382 (46.3)	192 (46.3)	190 (46.3)	0.982
Female	443 (53.7)	223 (53.7)	220 (53.7)	
Age				
60-69	418 (50.7)	207 (49.9)	211 (51.5)	0.677
70-79	277 (33.6)	138 (33.3)	139 (33.9)	
80 and above	130 (15.8)	70 (16.9)	60 (14.6)	
Marital status				
Married	600 (72.7)	325 (78.3)	275 (67.1)	< 0.001
Others^a^	225 (27.3)	90 (21.7)	135 (32.9)	
Educational level				
Primary school and above	445 (53.9)	216 (52.0)	229 (55.9)	0.273
Illiterate	380 (46.1)	199 (48.0)	181 (44.1)	
Problems in mobility				
Yes	135 (16.4)	71 (17.1)	64 (15.6)	0.561
No	690 (83.6)	344 (82.9)	346 (84.4)	
Problems in self-care				
Yes	67 (8.1)	36 (8.7)	31 (7.6)	0.558
No	758 (91.9)	379 (91.3)	379 (92.4)	
Problems in usual activities				
Yes	153 (18.5)	88 (21.2)	65 (15.9)	0.048
No	672 (81.5)	327 (78.8)	345 (84.1)	
Problems in pain/discomfort				
Yes	353 (42.8)	189 (45.5)	164 (40.0)	0.108
No	472 (57.2)	226 (54.5)	246 (60.0)	
Problems in depression/anxiety				
Yes	101 (12.2)	52 (12.5)	49 (12.0)	0.800
No	724 (87.8)	363 (87.5)	361 (88.0)	

^a^represents single, widowed and divorced

As to different dimensions of EQ-5D, old people who were left behind had higher proportion of problems in usual activities, while for other dimensions there was no differences between those left behind and not left behind.

### Dimensions and scores of social capital

Based on the factor analysis, fourteen social capital related questions were classified into different dimensions with Varimax rotation and an eigenvalue greater than 1. The KMO was 0.724 and the *p* value of Bartlett’s test was less than 0.001. The procedure yielded four dimensions and explained 67.3% of the total variance. The eigenvalue for four dimensions was 3.488, 2.856, 1.828 and 1.254 respectively and four dimensions accounted for 21.3, 17.7, 16.9 and 11.4% of the total variance respectively. Four dimensions were named as: *social participation*, *neighborhood relationship and support*, *trust and reciprocity* and *personal networks*. *Social participation* involved the frequency and quantity of organization participation. *Neighborhood relationship and support* was concerned with the relationship and mutual support between the neighbors. *Trust and reciprocity* included trust in community residents as well as mutual aid. *Personal networks* related to the number of friends and relatives and the occupations of these persons. Factor loadings are presented in Table 2. The reliabilities of the four identified constructs, presented as Cronbach’s alpha, were *α* = 0.871 for *social participation*, *α* = 0.717 for *neighborhood relationship and support*, *α* = 0.398 for *trust and reciprocity* and *α* = 0.711 for *personal network*. In general, the reliability and validity of the factor analysis were relatively good.

**Table 2:** Factor loadings of social capital measures

Questions	Factor loading			
	Social participation	Neighborhood relationship and support	Trust and reciprocity	Personal networks
1. Do you participate in some organizations?	**0.986**	0.009	0.022	0.072
2. How many organizations do you participate in?	**0.973**	0.003	0.020	0.054
3. Do you often participate activities in such organizations?	**0.969**	0.011	0.044	0.102
4. Do you trust majority villagers in your village?	−0.026	0.062	**0.839**	−0.009
5. Do you trust the leaders in your village?	0.067	−0.088	**0.840**	−0.149
6. Is there anyone who looks after you when you are sick?	0.015	0.279	**0.633**	0.232
7. How many relatives do have?	−0.061	0.110	−0.173	**0.742**
8. How many friends do you have?	0.040	0.131	0.247	**0.694**
9. What are the occupations of your relatives and friends who have a close relationship with you?	0.277	0.028	−0.010	**0.652**
10. Is there anyone who takes care of your house when you are absent?	−0.135	**0.549**	0.027	0.195
11. Do your neighbor help you when you are sick?	0.014	**0.624**	0.516	0.104
12. Are you willing to lend some money to your neighbor when they need?	0.100	**0.556**	0.442	0.011
13. Do you often chat with your neighbor?	0.054	**0.824**	0.022	0.039
14. Do you often visit your neighbor?	0.049	**0.822**	−0.014	0.040

Note: bold means these factor loadings are grouped into one dimension

Social capital score was calculated as following:

$$ \mathrm{Social}\kern0.5em \mathrm{capital}\kern0.5em \mathrm{score}=\frac{3.844}{3.488+2.856+1.828+254}\times \mathrm{F}1+\frac{2.856}{3.488+2.856+1.828+1.254}\times \mathrm{F}2+\frac{1.828}{3.488+2.856+1.828+1.254}\times \mathrm{F}3+\frac{1.254}{3.488+2.856+1.828+1.254}\times \mathrm{F}4 $$

### Social capital and HRQoL

Table 3 shows T-test analysis between EQ-5D index and each categorical variable, stratified by being left behind or not. Among the group being left behind, high social capital indicated higher EQ-5D index, while for those not being left behind, there was no difference. Among the group left behind, the differences of EQ-5D index between sex, age, marital status and educational level were also statistically significant. Older adults who were female, of higher age, illiterate and not married, scored lower in EQ-5D index within their respective category. Similarly, for those not left behind, higher age, being not married and illiterate were also associated with lower EQ-5D index. T-test analysis was also conducted to compare EQ-5D index among those left behind and not left behind. The EQ-5D index for the two subgroups were 0.860 and 0.879 respectively, but the difference was not significant.

**Table 3:** EQ-5D index by categorical variables, stratified by being left behind and not being left behind

Variables	Left behind			Not left behind		
	Mean	SD	*P* value	Mean	SD	*P* value
Social capital^a^						
High	0.880	0.143	< 0.001	0.885	0.135	0.162
Low	0.793	0.231		0.861	0.198	
Sex						
Male	0.886	0.144	0.004	0.888	0.152	0.269
Female	0.838	0.189		0.871	0.156	
Age						
60-69	0.869	0.179	< 0.014	0.915	0.120	< 0.001
70-79	0.874	0.149		0.877	0.136	
80 and above	0.806	0.180		0.754	0.225	
Marital status						
Married	0.873	0.162	< 0.004	0.903	0.136	< 0.001
Others^b^	0.814	0.194		0.830	0.178	
Educational level						
Primary school and above	0.887	0.139	0.001	0.905	0.140	< 0.001
Illiterate	0.831	0.196		0.846	0.166	

^a^the score of social capital was dichotomized into low (lowest 25% of the sample) and high (>25%) social capital

^b^represents single, widowed and divorced

Table 4 shows the results of Tobit regression between social capital and EQ-5D index, with coefficients and standard error. Model 1 included individual characteristics, social capital and being left behind or not. The negative coefficients suggested that those 80 years old and above, not married and illiterate had lower EQ-5D index. The EQ-5D index among those with low social capital was significantly lower (0.055) than among those with high social capital. The EQ-5D index among those left-behind was significantly lower (0.040) than among those not left behind. In model 2, different dimensions of social capital were included. The results indicated that only cognitive social capital *trust and reciprocity* had a significant association with EQ-5D index. In model 3, interactions between social capital and left behind were further included. The interaction was significant, which meant, that those who both had low social capital and were left behind, had lower EQ-5D index.

**Table 4:** Tobit regression of social capital and EQ-5D index (total sample)

Variables	Model 1		Model 2		Model 3	
	β	SE	β	SE	β	SE
Sex^a^						
Female	−0.019	0.021	−0.018	0.021	−0.020	0.021
Age^b^						
70-79	−0.019	0.022	−0.023	0.021	−0.016	0.022
≥ 80	−0.113***	0.029	−0.120***	0.028	−0.111***	0.029
Marital status^c^						
Others	−0.062**	0.023	−0.061**	0.022	−0.062**	0.023
Educational level^d^						
Illiterate	−0.071**	0.021	−0.073**	0.021	−0.069**	0.021
Social capital^e^						
Low	−0.055*	0.022			−0.013	0.031
Left behind^f^						
Yes	−0.040*	0.019			−0.019	0.022
Social participation^g^						
Low			−0.009	0.023		
Neighborhood relationship and support^h^						
Low			−0.018	0.022		
Trust and reciprocity^i^						
Low			−0.102***	0.022		
Personal networks^j^						
Low			0.035	0.023		
Social capital*left behind					−0.084*	0.044
Observations	825		825		825	
Pseudo R^2^	0.110		0.134		0.115	

^a^Reference: male

^b^Reference: 60-69 years old

^c^Reference: married

^d^Reference: primary school and above

^e^Reference: high social capital

^f^Reference: not left behind

^g^Reference: high social participation

^h^Reference: high neighborhood relationship and support

^i^Reference: high trust and reciprocity

^j^Reference: high personal networks

***p < 0.001; ** *p* < 0.01; * *p* < 0.05

### The difference between being left behind and not being left behind

In Table 5, the total sample was stratified between those left behind and not left behind. Tobit regressions of social capital and EQ-5D index were conducted in both subgroups. In all models, the coefficients of low social capital were negative. However, the association with EQ-5D index was significant only in the subgroup of being left behind. This suggests that, for those left behind, low social capital indicated lower EQ-5D index. Among those being not left behind, the association was not significant. In this subgroup, old age, being not married and illiterate were related to low EQ-5D index.

**Table 5:** Tobit regression of social capital and HRQoL (subgroup of being left behind and not being left behind)

Variables	Being left behind								Not being left behind							
	Model 1		Model 2		Model 3		Model 4		Model 1		Model 2		Model 3		Model 4	
	β	SE	β	SE	β	SE	β	SE	β	SE	β	SE	β	SE	β	SE
Sex^a^																
Female	−0.078**	0.028	−0.079**	0.027	−0.072*	0.028	−0.049	0.030	−0.024	0.027	−0.024	0.027	−0.019	0.027	0.009	0.028
Age^b^																
70-79	0.005	0.031	0.013	0.031	0.018	0.031	0.021	0.031	−0.076*	0.030	−0.076*	0.030	−0.058	0.030	−0.054	0.030
≥ 80	−0.091*	0.038	−0.072	0.038	−0.051	0.040	−0.038	0.041	−0.240***	0.038	−0.239***	0.038	−0.206***	0.040	−0.195***	0.040
Social capital^c^																
Low			−0.108**	0.032	−0.111**	0.032	−0.108**	0.032			−0.005	0.030	−0.009	0.030	−0.013	0.030
Marital status^d^																
Others					−0.053	0.035	−0.051	0.035					−0.074*	0.030	−0.059*	0.030
Educational level^e^																
Illiterate							0.055	0.030							−0.081**	0.029
Observations	415		415		415		415		410		410		410		410	
Pseudo R^2^	0.045		0.079		0.086		0.096		0.123		0.124		0.143		0.167	

^a^Reference: male

^b^Reference: 60-69 years old

^c^Reference: high social capital

^d^Reference: married

^e^Reference: primary school and above

***p < 0.001; ** *p* < 0.01; * *p* < 0.05

## Discussion

### Key findings of our study

This study explored the association between social capital and HRQoL among older people in rural China. We found that among old people who were left behind, low social capital was significantly associated with low HRQoL. The main effects came from cognitive social capital (i.e. *trust and reciprocity*). The results from this study suggest that, left behind old people with low social capital is a potentially vulnerable group in rural China.

### Social capital and HRQoL

Our results are in line with other studies indicating associations between social capital and HRQoL [10, 22, 35, 36]. According to Putnam (2000) [37], the association may be explained by the fact that individuals who are less socially engaged and trusting are more easily ignored, socially isolated, more stressed and as a result, are less healthy.

Concerning the different dimensions of social capital, we found that cognitive social capital *trust and reciprocity* exhibited positive associations with HRQoL. *Trust and reciprocity* refers to norms and attitudes including trust, shared values and mutual aid. Similar results have been found in another study from rural China showing that cognitive social capital was positively associated with health outcomes while little statistical association existed between structural social capital and health outcomes [16]. It might be interpreted that, cognitive aspects of social capital could increase self-esteem, self-satisfaction and confidence in the older population and then influence health outcomes [38]. Compared to other studies in China, we did not find any significant association between structural social capital such as social participation and HRQoL [31]. As Sun pointed out, few older people in rural China participate in social activities or formal organizations. Instead, they prefer informal activities. Hence, our measures of social participation may underestimate this kind of effects on HRQoL [31]. Therefore, the importance of different dimensions of social capital should be assessed because they may not be related to health outcomes to the same degree [15].

### The difference in HRQoL between those left behind and not left behind

When looking at the whole sample, there was no significant association between being left behind and HRQoL in the present study. Previous studies have shown ambiguous results regarding the effect of children’s migration on left behind older people. Some studies showed negative consequences while others showed positive effects [39, 40]. Previous studies have reported lower depression symptoms among left-behind elderly or no difference between the two groups [32]. One interpretation of our results could be that, as some researchers point out, children’s migration contributes to the material well-being of their parents and the better economic status is associated with less adverse health outcomes. Negative effects of migration may also be attenuated by new communication technology, which increases the possibilities for “caring at distance”, and transportation improvements in rural areas [39, 41]. Another possible explanation is linked to the geographical selection of the sample. Our survey was conducted in Jiangsu province, which represents a wealthy area in eastern China, where the distance of migration to cities was shorter than in western areas. Most of the adult children migrated to nearby cities. They could often go back and visit their left behind parents. Even though they stayed in cities for more than six months per year (according to the definition of being left behind), their frequent visits could compensate the adverse effects of being left behind.

The association between social capital and HRQoL was different among those being left behind and those not left behind. For those who were not left behind, there was no difference in HRQoL, whether they had high or low social capital. This may indicate that, reverse causality (i.e. that children of parents in poor health might be less likely to migrate) might exist among this group. Giles and Mu (2007) found that parents’ health affects children’s migration decision in China [42]. If older parents have poor health, their children will reduce migration to cities [43]. Hence, parents can live with their children and get more support from them, so social capital such as neighborhood support, trust and reciprocity and social networks may not be important resources of their HRQoL. In the present study, low social capital was significantly associated with low HRQoL for those who were left behind. That is to say, the left behind older people with low social capital can be identified as a vulnerable group in rural China.

### Strengths and limitations of the study

A strength of this study is the comparatively large sample size and high response-rate. Before the survey the interviewers introduced themselves and the questionnaires to the respondents and got their support and cooperation. Another strength is that the study could distinguish between those being left behind or not, linking responses concerning social capital and HRQoL, and permitting analysis of the association between these in both groups.

Some limitations should be stated. First, the association between social capital and HRQoL may indicate reverse causality. Those who had low HRQoL may have reduced social participation, neighborhood relationship and support because of their low HRQoL. Likewise, adverse health conditions of older parents can directly impinge on their children’s migration decisions and then keep children around them. Due to the cross sectional design, we cannot rule out reverse causality. To confirm causality, there is a need for longitudinal studies. A second limitation was that, our survey was only conducted in Jiangsu Province, an economically advanced coastal area. The sample included only three counties in one province. Hence, we cannot draw any general conclusions and more regions should be included in future studies to increase generalisability. Thirdly, our study mainly focuses on individual social capital, we did not consider the community level of social capital. Finally, the internal consistency reliability of the dimension *trust and reciprocity* was relatively low, which may underestimate the effects on HRQoL of this dimension.

### Implications

In China, the massive exodus of young adults to cities will continue and the increase in the number of left-behind older people is inevitable [42]. Care and well-being of older people left behind has become a major public health challenge in China. Older people have long been described as a vulnerable group in several respects. The basic living conditions are tough in many parts of rural China. Old people have extremely limited access to pensions or other state support and most of them are dependent on their children. This study identified left behind older people with low social capital as a potentially vulnerable group. Given the inadequate institutional support from rural governments, old people being left behind must turn to their families for financial support, physical care and psychological help to maintain their health. Reliable and feasible measures such as enhancing social capital could be beneficial for old people who were left behind. Further detailed studies on the living conditions of left behind older people in China could provide the basis for policies, programs, decisions and future planning on how to best take care of and protect one of society’s most vulnerable groups.

## Conclusions

This study suggests that low social capital was significantly associated with low HRQoL among old people who are left behind. The left behind old people with low social capital are a potential vulnerable group in rural China. Formulating and implementing initiatives and strategies which increase social capital may foster better HRQoL, especially for old people who were left behind.

## Additional file

Additional file 1: Social capital related questions. (DOCX 14 kb)

## Abbreviations

EQ-5D: European quality of life-5 dimensions
HRQoL: Health-related quality of life
SD: Standard deviation
SE: Standard error
SF-36: The short-form health survey
