# Supplementary material for: Association between social capital and health-related quality of life among left behind and not left behind older people in rural China
Source: BMC Geriatr. 2017 Dec 16;17:287. doi: 10.1186/s12877-017-0679-x (PMC5732484; doi:10.1186/s12877-017-0679-x)
Supplement: Additional file 1: — Social capital related questions. (DOCX 14 kb) [file 12877_2017_679_MOESM1_ESM.docx]

Additional file 1: Social capital related questions

| Items | | Questions |
| --- | --- | --- |
| 1  2  3 | Do you participate in some organizations?  How many organizations do you participate in?  Do you often participate activities in such organizations? | |
| 4  5  6 | Do you trust the majority of villagers in your village?  Do you trust the leaders in your village?  Is there anyone who looks after you when you are sick? | |
| 7  8  9 | How many relatives do you have?  How many friends do you have?  What are the occupations of your relatives and friends who have a close relationship with you? | |
| 10  11  12  13  14 | Is there anyone who takes care of your house when you are absent?  Does your neighbor help you when you are sick?  Are you willing to lend some money to your neighbor when they need?  Do you often chat with your neighbor?  Do you often visit your neighbor? | |
